# Partial response in a patient with skeletal and hepatic metastases following resected pancreatic cancer to the novel cell therapy SL-28: a case report

**DOI:** 10.3389/fonc.2025.1636989

**Published:** 2025-11-19

**Authors:** Victor Tetz, Kristina Kardava, Olzhas Shulenbayev, Maria Vecherkovskaya, Alireza Khodadadi-Jamayran, Aristotelis Tsirigos, George Tetz

**Affiliations:** 1 Department of Structural Biology, Human Microbiology Instititue, New York, NY, United States; 2 Private Hospital International Almaty, Almaty, Kazakhstan; 3 Applied Bioinformatics Laboratories, Division of Advanced Research Technologies, New York University (NYU) School of Medicine, New York, NY, United States; 4 Department of Pathology, New York University School of Medicine, New York, NY, United States; 5 Department of Medicine, Division of Precision Medicine, NYU School of Medicine, New York, NY, United States; 6 Tetz-Laboratories, New York, NY, United States; 7 Second Life Therapeutics, New York, NY, United States

**Keywords:** pancreatic cancer, bone metastasis, liver metastasis, oncology, cell therapy, leucocyte-tells, TezR, SL-28

## Abstract

Pancreatic cancer is a deadly and highly metastatic malignancy; the liver is the most frequent site of metastasis (70-80%), however, bone metastases are rare, occurring in approximately 5% of cases. Currently, there are no documented reports of effective responses to therapy for bone metastases, especially in the context of cell-based treatments. Here, we report the first case of a partial response according to RECIST v1.1, with a reduction in size and dissolution of liver and bone metastases from pancreatic cancer, for the novel cell therapy, SL-28, under the expanded access pathway (NCT06872489). SL-28 (Leukocyte-Tells) is a novel cell therapy that uses allogeneic leukocytes, whose anticancer activity is increased *ex vivo* using the recently discovered Universal Receptive System. A 79-year-old female, staged T4N0M1 after distal pancreatectomy with splenectomy, developed liver and bone metastases that were unresponsive to chemotherapy. A partial response was achieved in the third month of monotherapy SL-28 with partial disappearance of the metastasis in the right femur and a reduction in the metastatic bone mass in the left pubic bone, with over a 30% decrease in the sum of the diameters of the target lesions. In the liver, some metastatic lesions disappeared along with a shrinkage in the size of others. SL-28 therapy was well tolerated with no serious adverse effects. This is the first clinical case describing the partial response of a patient with liver and bone metastases after resection of pancreatic cancer to cell therapy using a novel type of cell therapy with SL-28.

## Introduction

Pancreatic cancer is one of the deadliest cancers, predominantly owing to late diagnosis, aggressive biology and rapid metastasis (1). While the typical sites of metastases in pancreatic cancer are the liver and peritoneum, bones are less common sites, representing approximately 5% of all cases of metastatic burden in patients with pancreatic cancer, and are considered poorly responsive to any therapy (2, 3).

Although adoptive cell-based immunotherapies are promising for the treatment of hematological malignancies and melanoma, their efficacy in solid tumors remains limited (4, 5). Adoptive cell therapies using chimeric antigen receptor (CAR) T cells and tumor-infiltrating lymphocytes (TILs) have generally shown limited success for the treatment of pancreatic cancer, and there have been no reported cases of bone metastases in response to these therapies (6). Here, we describe an interesting case of a partial response, according to RECIST 1.1, for both visceral and bone metastases to a novel type of cell therapy with Leukocyte-Tells (SL-28).

SL-28 is an allogeneic (HLA-mismatched) cell-based therapy comprising leukocytes (lymphocytes and granulocytes) obtained from the blood of healthy donors using double gradient centrifugation with Histopaque 1,077 and 1,119 (7, 8). Leukocytes in SL-28 exhibit enhanced activity and altered immunogenicity, orchestrated through the Universal Receptive System without any direct genetic modification, and are referred to as Leukocyte-Tells. They represent a completely different type of cell therapy that is not related to chimeric antigen receptor T cells, tumor-infiltrating lymphocytes, or any other cell therapy (9–11). Unlike CAR-T cells, whose anticancer activity is enhanced through gene editing to recognize and eliminate cells expressing a specific antigen, or TIL therapy, in which a patient’s tumor-infiltrating lymphocytes are expanded outside the body and reinfused to attack the patient’s tumor, Leukocyte-Tells are neither genetically modified nor trained against a particular tumor type (12). Increased antitumor activity was achieved through novel regulatory pathways triggered by the destruction of teazeled receptors (TezRs). Functional pathway analysis revealed that over 1,300 upregulated, differentially expressed genes were enriched in four broad functional categories: immune cell signaling, immune cell migration, membrane uptake, and enhanced energy metabolism (8).

Notably, this is the fourth clinical case describing the effects of SL-28 in patients with different types of advanced solid tumors (in press).

Access to and use of investigational cell therapies for patients with advanced solid tumors vary across countries, with the allowance to use them based on local IRB approval in the Eurasian Union, including Kazakhstan. The compassionate use (expanded access) pathway was employed under the NCT06872489 standard.

## Case presentation

In November 2024, a 79-year-old female patient presented with a chief complaint of metastatic liver disease that was unresponsive to chemotherapy. The patient was diagnosed with pancreatic adenocarcinoma pT3N0M0 in 2023 and underwent a distal pancreatectomy and splenectomy in May 2023. In August 2023, the patient was diagnosed with multiple liver and bone metastases. Due to her poor performance and nutritional status, the patient was not considered a candidate for surgical resection.

The patient received capecitabine (1000 mg/m^2^, BID) from August 2023 till January 2024, followed by seven cycles of chemotherapy with capecitabine plus oxaliplatin (XELOX) (13). Next, she received another course of capecitabine (1000 mg/m^2^, BID) from July 2024 to October 2024. The patient tolerated therapy poorly, experiencing vomiting and nausea. Due to the lack of a therapeutic response, chemotherapy was discontinued, and the patient was left only with supportive therapy with tramadol.

Because of a lack of response to existing therapy, experimental SL-28 therapy was recommended. The administration of SL-28 was approved by the Institutional Review Board (#246), and the study was conducted in accordance with the NCT06872489 and the approved protocol. No commercial sponsors were involved in this study.

Upon examination in November 2024, laboratory results showed an increased CA 19–9 of 8420 U/mL, D-dimer level of 1200 ng/mL fibrinogen equivalent units (FEU), ferritin level of 950 ng/mL, and lactate dehydrogenase (LDH) of 510 U/L. Total bilirubin, alanine aminotransferase (ALT), and aspartate aminotransferase (AST) at 2.63 mg/dL, 85 U/L, and 92 U/L, respectively, were all elevated over normal limits (**Table 1**). The blood albumin level was below normal at 2.8 g/dL.

**Table 1:** Laboratory Parameters at Baseline and During Treatment.

Parameter	Baseline	Day 30	Day 70
CA 19-9	8420 U/mL	4256 U/mL	1547 U/mL
D-Dimer	1200 ng/mL FEU	874 ng/mL FEU	790 ng/mL FEU
Ferritin	950 ng/mL	740 ng/mL	487 ng/mL
LDH	510 U/L	312 U/L	278 U/L
Total bilirubin	2.63 mg/dL	2.05 mg/dL	2.11 mg/dL
AST	85 U/L	56 U/L	56 U/L
ALT	92 U/L	91 U/L	52 U/L
Total protein	5.9 g/dL	6.3 g/dL	5.5 g/dL
Albumin	2.8 g/dL	3.6 g/dL	3.8 g/dL
Creatinine	1.24 mg/dL	1.17 mg/dL	1.09 mg/dL
Urea	26.6 mg/dL	23.2 mg/dL	22.7 mg/dL
RBC	3.40 x 10^6/µL	3.70 x 10^6/µL	3.80 x 10^6/µL
Hemoglobin	9.4 g/dL	9.8 g/dL	10.2 g/dL
Platelets	460 x 10^3/µL	456 x 10^3/µL	440 x 10^3/µL
WBC	11.8 x 10^3/µL	10.6 x 10^3/µL	9.4 x 10^3/µL
Lymphocytes	10%	12%	15%
ESR	52 mm/hr	45 mm/hr	35 mm/hr

**Figure 1:**
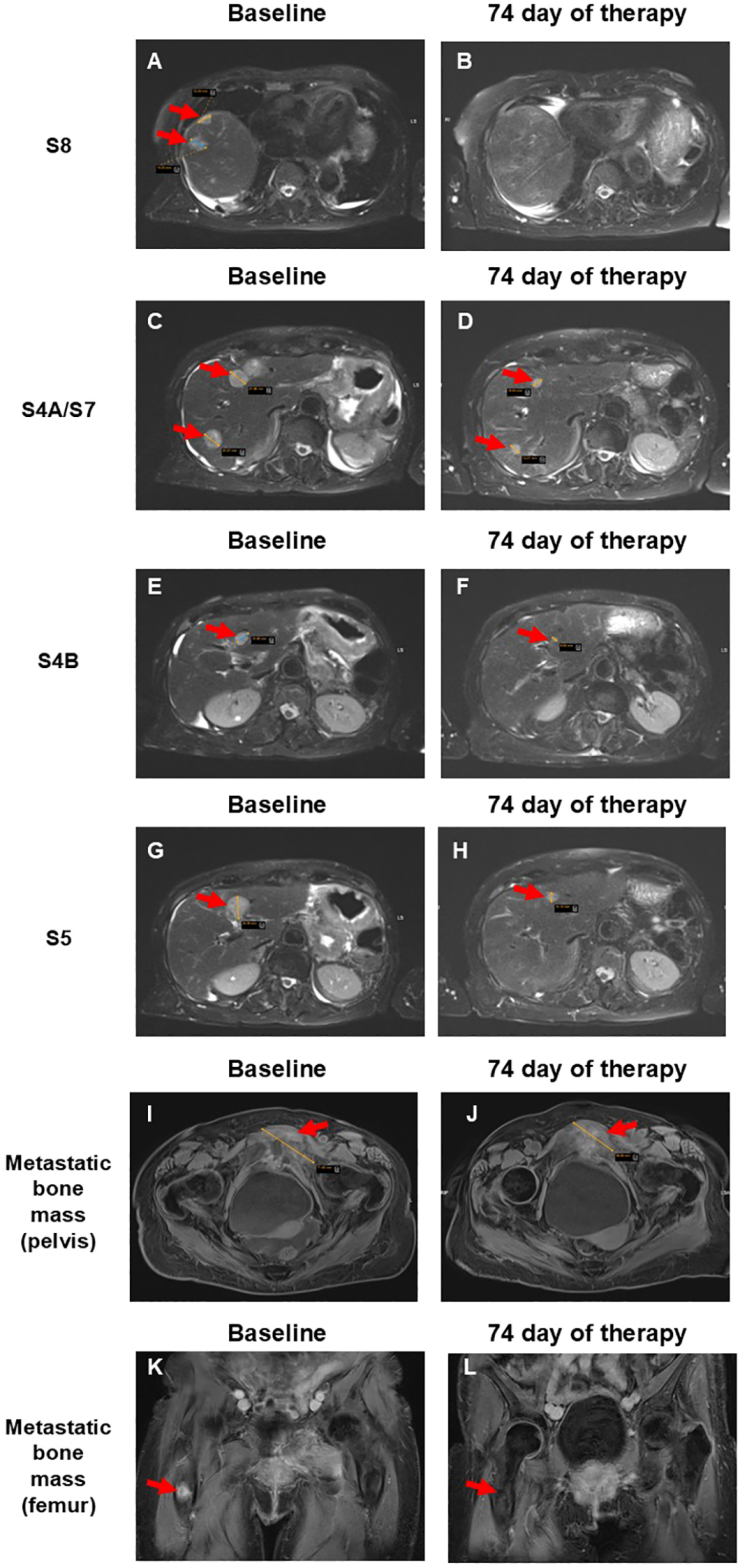
Comparison of pre- and post-therapy MRI scans of a patient with liver and bone metastases from pancreatic cancer after copocaudalic resection following SL-28 therapy. **(A, B)** Disappearance of two lesions measuring 2.0 and 1.5 cm in the S8 segment. **(C, D)** Reduction of lesions in the S4A and S7 segments from 2.5 and 2.2 cm to 1.0 and 1.1 cm, respectively. **(E, F)** Segment 4B lesion decreased from 1.5 cm to 0.5 cm. **(G, H)** Reduction of the lesion in S5 from 2.3 cm to 1.7 cm. **(I, J)** Metastatic bone mass near the left pubic bone decreased from 7.2 to 5.7 cm. **(K, L)** Disappearance of metastatic bone mass in the right femur. Red arrows indicate metastatic lesions.

Further diagnostic evaluation included magnetic resonance imaging (MRI) (**Figure 1**), which detected multiple metastatic masses in liver segments S4A, S4B, S5, S7, and S8. Blastic lesions were also observed in the femur and pubic bones.

SL-28 treatment was initiated in November 2024, starting at a low dose of 1 × 10^7 cells per injection and gradually escalating to 1 × 10^8, administered once or twice daily, from 2 times a week up to 7 times a week. The infusions, cryopreserved prior to administration, were delivered to the patient over 2 weeks.

SL-28 therapy was well tolerated, even at the highest dose, with no signs of cytokine release syndrome, graft-versus-host disease, or immune effector cell-associated neurotoxicity syndrome. A mild fever with a maximum temperature of 37.7°C was episodically noted within 3 h post SL-28 injection, lasting up to 4 h, and was self-limiting.

After the first 30 days of treatment, positive changes in blood markers indirectly associated with tumor progression were observed (**Table 1**). CA 19–9 reached 4256 U/mL (baseline 8420 U/mL), D-dimer level reduced from 1200 mg/mLto 874 ng/mL FEU, ferritin was reduced from 950 to 740 ng/mL, and LDH decreased to 312 U/L. Total bilirubin remained elevated at 2.05 mg/dL, as did AST at 65 U/L; however, both showed a downward trend. Simultaneously, ALT remained largely unchanged - 91 U/L (baseline 92 U/L) (**Table 1**). Albumin, initially below the normal range at baseline, returned to normal levels, increasing to 3.6 g/dL.

Based on these positive pharmacodynamics, therapy was continued. On the 70th day of therapy, laboratory analysis confirmed the same positive trend (**Table 1**). CA 19–9 reached 1547 U/mL, D-dimer reduced to 790 ng/mL FEU, ferritin decreased to 487 ng/mL, and LDH decreased to 278 U/L. Furthermore, total bilirubin was 2.11 mg/dL, ALT 52 U/L, and AST 56 U/L. Albumin showed a slight increase to 3.8 g/dL.

An MRI performed 74 days after initiation of SL-28 therapy revealed similar positive pharmacodynamics. Both lesions in liver segment S8 (previously 2.0 and 1.5 cm) had completely disappeared (**Figures 1A, B**). The segment 4A lesions, initially measuring 2.5 cm and 2.2 cm, were reduced to 1.0 cm and 1.1 cm, respectively. The lesion in S7 showed a marked decrease in size, now measuring 1.1 cm (baseline, 2.0 cm) (**Figures 1C, D**). The segment 4B lesion decreased from 1.5 cm to 0.5 cm (**Figures 1E, F**), and the lesion in S5 was reduced from 2.3 cm to 1.7 cm (**Figures 1G, H**). The observed > 30% decrease in the sum of the diameters of target lesions corresponds to the partial response according to RECIST 1.1. criteria (14). The metastatic bone mass near the left pubic bone and pubic symphysis decreased from 7.2 cm to 5.7 cm and appeared more necrotic, with increased changes in adjacent soft tissue and muscle (**Figures 1I, J**). The lesion detected prior to SL-28 therapy in the proximal right femur was now barely visible (**Figures 1K, L**). This corresponds to a partial response of bone metastases according to the MD Anderson criteria (15). The patient continues SL-28 therapy.

## Discussion

Here, we report a partial response according to RECIST 1.1 criteria following treatment with SL-28 in a patient with chemotherapy-resistant liver and bone metastases after distal pancreatectomy with splenectomy.

Bone metastases in pancreatic cancer are less common than visceral metastases. The uniqueness of this case lies in the partial response of both osteoblastic (pelvic and femoral) and hepatic metastases to a novel first-in-class cell therapy. This is particularly noteworthy, as pancreatic cancer is generally considered poorly responsive to chemotherapy as well as immune- and cell-based therapies (16). At the time of initiating SL-28 therapy, our patient had been unresponsive to chemotherapy and was receiving only supportive analgesic treatment.

SL-28 is a novel form of allogeneic cell therapy, in which the activity of donor-derived white blood cells is increased through the newly identified Universal Receptive System to generate so-called “Leukocyte-Tells,” through the modification of TezR receptors as previously described (8, 9). In earlier studies, leukocytes demonstrated activity against various tumor types both *in vitro* and *in vivo* (7, 8, 17). Specifically, Leukocyte-Tells have been shown to have multiple antitumor mechanisms that are upregulated in naïve white blood cells. Leukocyte-Tells showed enhanced anticancer and antimicrobial bioactive compound (AABCs) production, with over 700 unique or differentially produced peptide and non-peptide metabolites that showed >100,000-fold higher activity than AABCs derived from control leukocytes (7). Second, the antimicrobial and anticancer activities of Leukocyte-Tells exceeded those of control leukocytes, and they showed up to 1,000,000-fold higher activities against a broad variety of microorganisms and cancer cell lines owing to greater phagocytic activity, production of granule enzymes, and enhanced cytokine production. Finally, gene expression analysis of Leukocyte-Tells revealed enrichment of genes associated with numerous functional pathways, including cell membrane uptake, and immune cell signaling. In particular, genes involved in immune cell migration were also enriched with *ESAM*, *JAM3*, *RAP1GDS1*, and *RAPGEF* being upregulated, suggesting that Leukocyte-Tells might have a high capacity to migrate toward tumors (18, 19). Taken together, these data suggest that SL-28 has a superior antitumor effect in patients. In expanded access studies, SL-28 exhibited high activity, inducing partial or complete responses in patients with advanced solid tumors, including neuroblastoma, prostate cancer, and lung cancer (in press 20).

The patient tolerated SL-28 therapy well during the study, despite numerous injections throughout the treatment course. No severe adverse effects were observed. CRS was systematically monitored according to ASTCT consensus criteria, including daily assessment for fever, hypotension, and hypoxia. No signs of CRS of any grade were observed. GvHD and ICANS were also monitored clinically (skin, gastrointestinal, hepatic, and neurological assessments) throughout the study, and no manifestations were detected. Cell blood and blood biochemical analyses were performed, and no significant abnormalities were identified (**Table 1**).

The study is still ongoing with the patient remaining on SL-28 monotherapy. However, by the third month of therapy, the patient had already shown remarkable improvement, with a reduction and partial disappearance of liver and bone lesions. Simultaneously, owing to the limitations of the single case study, additional clinical research is required to fully uncover the effect of SL-28 in cancer patients, particularly in identifying the responding and non-responding patient populations.

Our early findings underscore the potential of SL-28 therapy and support further investigations in patients with different tumor types, requiring full-scale multicenter clinical trials.

## Data Availability

The original contributions presented in the study are included in the article/Supplementary Material. Further inquiries can be directed to the corresponding author.
